# Supplementary material for: Broadening the Taxonomic Breadth of Organisms in the Bio-Inspired Design Process
Source: Biomimetics (Basel). 2023 Jan 23;8(1):48. doi: 10.3390/biomimetics8010048 (PMC9944075; doi:10.3390/biomimetics8010048)

**Supplementary Table S1: Schedule of Class Topics.** Students had to complete 10 of 11 pre-class brainstorming assignments focused on the bolded topics shown below. For each week of the course, students brainstormed systems relevant to a problem prior to class, and the class was spent reviewing basic research in animal behavior relevant to that problem. The syllabus contained the following language about these assignments: *"Weekly class preparation (150 points, 15%): Each week, you will be asked to prepare for lecture discussions with a "brainstorming activity." We will be focused on a "problem," and you will be asked to brainstorm some biological systems that offer an analogy for solving this problem (e.g., "making an agile robot makes me think of studying insect flight behavior). You will be asked to find a relevant paper from the primary literature and comment on some aspect of that paper. This is the only pre-class preparation required (all content will be delivered in class)."*

| Date | Class topic (problem: key concepts) |
|---|---|
| 20-Jan | Introduction to course |
| 25-Jan | Structure of science: confirmation bias, applied-basic |
| 27-Jan | Intro to data, statistics, and projects |
| 1-Feb | **Force**: adaptation, biomechanics |
| 3-Feb | **Force**: ecology and evolution cont., more stats |
| 8-Feb | **Robotics**: sensory systems and trait origins |
| 10-Feb | **Robotics**: neural processing |
| 15-Feb | **Anxiety/fear**: sensory biases, nature/nurture |
| 17-Feb | **Grief and depression**: proximate/ultimate approaches |
| 22-Feb | **Memory**: learning, neural plasticity |
| 24-Feb | **Memory**: evolution of cognition |
| 1-Mar | **Play, fun and music**: behavioral development |
| 3-Mar | **Play, fun and music**: some more statistics |
| 8-Mar | Exam 1 |

| | |
|---|---|
| 10-Mar | Statistics II |
| 15-Mar | **Healthy choices, food**: traps and nudges, optimal foraging |
| 17-Mar | **Healthy choices, movement**: habitat choice, landscape of fear |
| 22-Mar | Statistics III |
| 24-Mar | **Infertility**: sexual seleciton and sperm competition |
| 29-Mar | **Manipulating light**: female choice, signals of quality |
| 31-Mar | **Manipulating light**: mating systems, parental care |
| Break! | |
| 12-Apr | Statistics IV |
| 14-Apr | **Social problems**: social interactions, groups |
| 19-Apr | **Communication**: noise, signals in environments |
| 21-Apr | **Communication**: honest signaling |
| 26-Apr | **Cooperative societies**: game theory and cooperation |
| 28-Apr | **War and Peace**: game theory and aggression |
| 3-May | Conclusions and Exam II |
| 12-May | Mini-conference (1:30-3:30 pm) |

**Supplementary Table S2. List of pre-class brainstorming prompts for the first set of topics.**

| | Due Date | Problem Prompts |
|---|---|---|
| Force | Jan 28 | **Brainstorming:** *This week we are considering the problem of "generating force" -- perhaps for a machine crushing things, or a robot designed for a new application. Brainstorm a list of animals, and/or animal traits, that you might look to for inspiration in solving some aspect of this problem. Please put each idea on a separate line.*<br>**Reflections:** *Please write a few sentences about your brainstorming list. How did you come up with these ideas? Which one do you think is the most promising to pursue and why? (long essays not needed -- just a few thoughts)*<br>**Next steps:** *For your most promising idea, please find a paper/article with more information on this animal, system, or trait. List the reference here. You are welcome to read through it as well, but for this first assignment, you just need to find a reference and we will discuss more in class.* |
| Mental Health | Feb 11 | **Brainstorming:** *Choose one of these two topics related to mental health: grief and depression OR anxiety and fear. Brainstorm a list of animals you might study for biological inspiration related to these questions. Write a few sentences explaining how you came up with this list.*<br>**Next steps:** *For one of the animals on your list, find a paper that addresses something related to that animal and the behavior relevant to your brainstorming (e.g., fear). Either screen-shot or copy and paste the abstract from this paper into a document and annotate with at least one thing you find effective about the abstract and one thing that could be improved.* |
| Healthy choices | Mar 4 | **Brainstorming:** *Choose one of the following aspects of healthy choices: food and nutrition OR activity and movement. Brainstorm a list of animals you might study for inspiration related to these aspects of human health. Write a brief paragraph discussing how you came up with this list, reflecting on how/whether you felt creative.*<br>**Next steps:** *Choose one of the following aspects of healthy choices: food and nutrition OR activity and movement. Brainstorm a list of animals you might study for inspiration related to these aspects of human health. Write a brief paragraph discussing how you came up with this list, reflecting on how/whether you felt creative.* |

**Supplementary Table S3.** ***Assignment prompts for intervention comparisons***. We varied the intervention type and the topic in a fully factorial manner to avoid any confounding effects of topic on how well the intervention works. Students were split alphabetically by their last name. These were the last two brainstorming assignments of the semester, due in late April.

| | **Outdoor prompt.** *Many of you have noted that you are struggling to think of new ideas. This week, we are going to try a "space sampling" activity. Imagine a "landscape of ideas" (see image) sparked by different biological systems. You can explore this landscape by considering species from across the tree of life (evolutionary space) or species from across different ecosystems and ecoregions (ecological space). Some of the ideas inspired by these systems might be awesome ("b" in image), but sometimes we are distracted by previous knowledge and get "stuck" on another idea ("a" in image). To increase our chances of finding a good idea, we can push ourselves to "sample" more of this space. The more we sample the greater the chance of finding a good idea. In addition, the greater diversity of systems we consider, the greater the range of different underlying mechanisms we can learn from.* In this activity, consider THE PROBLEM. *What biological systems come to mind for inspiration related to this problem? Write down your first ideas, then push yourself to consider systems in other parts of ecological and evolutionary space. For example, go to http://tolweb.org/tree/ and hit "random page" to consider another lineage or navigate through taxonomic groups in Wikipedia (e.g., navigate to a different class). Or consider systems in different ecoregions https://en.wikipedia.org/wiki/Listof_terrestrial_ecoregions(WWF). Use this exploration to broaden your systems considered, then circle your favorite idea. Upload a picture or pdf.* | **Idea-Space prompt**. *Many of you have noted that you are struggling to think of new ideas. This week, we are going to try an "outdoor wandering." Find a place to walk outside for 30 minutes -- down your street, in a park, wherever -- and watch animals and nature. As you are wandering, consider THE PROBLEM. In your wandering, what biological systems do you see that make you think of ideas for addressing the problem of communication. Draw a little diagram or sketch of your wanderings, labeling the biological systems you noted along the way. Upload a picture or pdf.* |
|---|---|---|
| ***Communication***. Consider the problem of "communication" -- maybe you want to increase reliability or honesty of communication, between people, online, in the media. | Due 4/15 for students in the first half of the alphabet. | Due 4/15 for students in the second half of the alphabet. |
| ***Cooperation***. consider the problem of "cooperation" -- | Due 4/22 for students in the second half of alphabet. | Due 4/22 for students in the first half of the |

| | | |
|---|---|---|
| maybe you want people to collaborate effectively, conform to mask mandates, or just be nice to each other in the grocery store… | | alphabet. |

**Supplementary Table S4. Student reflections on the “idea-space” and “outdoor” interventions**

| **Outdoor** | **Idea-space** |
|---|---|
| *I thought this exercise was fun to do. I like going outside, but I usually don't have a reason to because I'm stuck at home with all of the schoolwork. In terms of the creativity here, it obviously depends on what I'm seeing,* | *The random page resource offered was a little difficult to navigate, and it was difficult to use because I didn't have much information on the animals that did show up. I got all birds, and it's hard to know whether or not they are good models for cooperation if I don't know anything about them.* |
| *I did stop a few times to sit and watch animals go about their business, but I thinking my best brainstorming happened when I was actively walking. Walking provided the right amount of activity to occupy my body, and allowed my mind to wonder. In addition, I got new inspiration every now and then form organism I came across in the environment* | *I liked the random page feature because it helped me to think of different animals or ecosystems when I got stuck. For example, an information page on squid appeared from the random page generator and made me think of the coral reef. I felt like this activity helped me to be more creative. I felt the most creative when a random page helped trigger a new direction in my thought process.* |
| *Most of the times I do these assignments cooped up in my room and I am mostly just ransacking my brain. However, going out to the park really allowed me to be more attentive.* | *I used the tree of life website and went to random pages, however almost all the random pages opened were insects. … I also went through the different clades of animals we covered in my animal diversity class and thought about what they may use for communication.* |
| *I got most of my ideas at random points, when something I noticed in the environment led my thoughts a certain way.* | *I felt like I got more caught up in what the difference was between the ecological and evolutionary spaces rather than focusing on brainstorming* |
| *I actually think this assignment kind of narrowed my think and made me a little less creative because I was only focusing on the organisms that were in front of me and not really any other ones. I got most of my ideas at random points, when something I noticed in the environment led my thoughts a certain way.* | *I did notice that my initial ideas were all mammals and insects, so the evolutionary space exploration helped me find different taxa of animals to explore. I did feel limited in the variety of taxa I was able to explore based on my familiarity with the species* |

**Supplementary Figure S1.** ***Image associated with the "idea-space" prompt***. See Table S3 for associated text with the assignment.

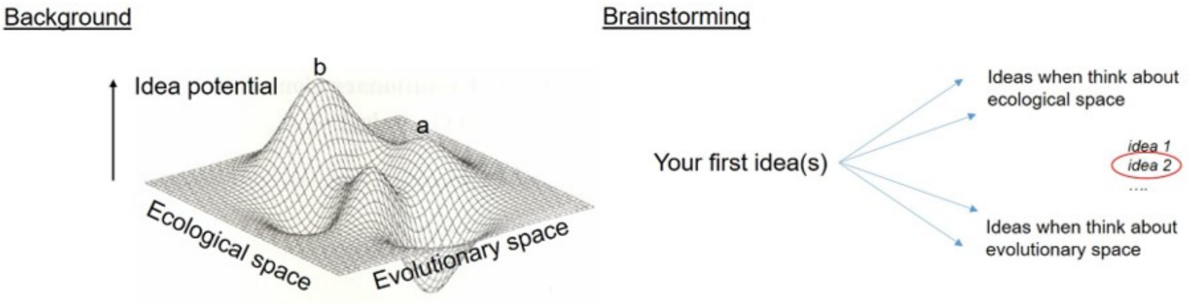


**Supplementary Figure S2. Distribution of student evaluation data between semesters where the course was problem-based (2021) versus a more traditional format (2018).**

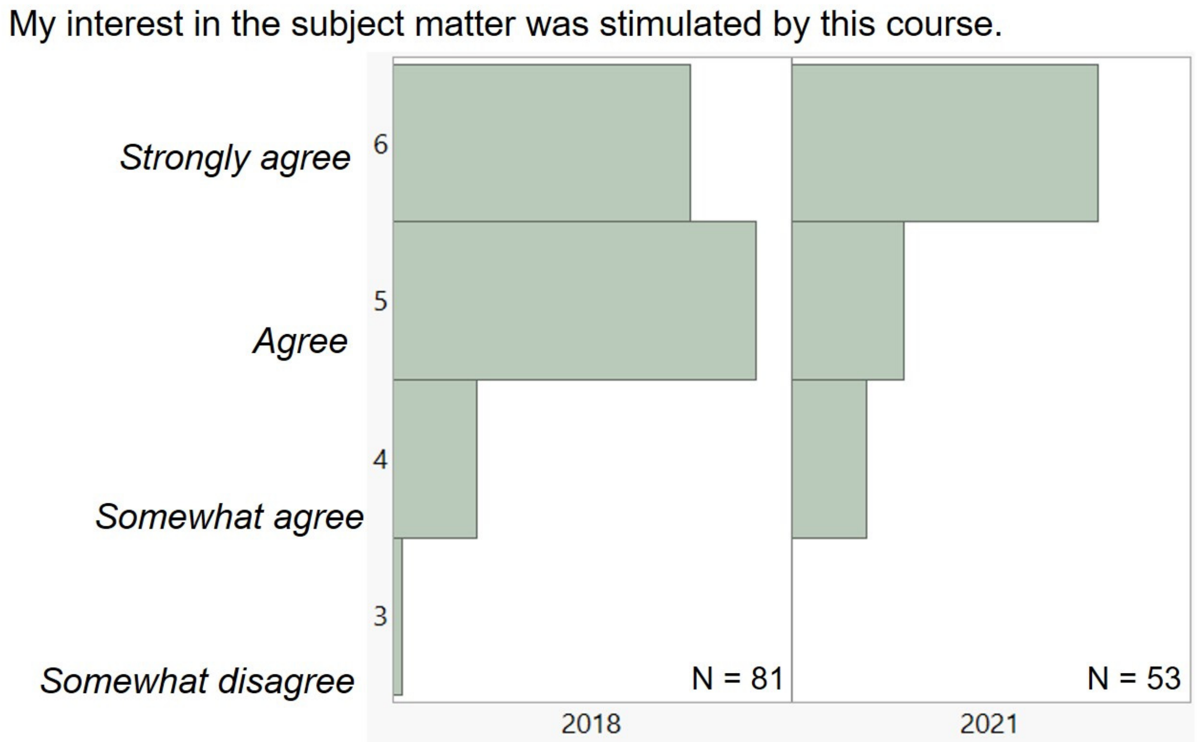

Supplement: Supplementary file 1 [file biomimetics-08-00048-s001.zip › biomimetics-2101198-supplementary.pdf]
